# Can acceptable quality angel food cakes be made using pasteurized shell eggs? The effects of mixing factors on functional properties of angel food cakes

**DOI:** 10.1002/fsn3.911

**Published:** 2019-02-10

**Authors:** Ajaypal Singh, David J. Geveke, Deana R. Jones, Eric D. Tilman

**Affiliations:** ^1^ Food Safety and Intervention Technologies Research Unit Eastern Regional Research Center Agricultural Research Service U.S. Department of Agriculture Wyndmoor Pennsylvania; ^2^ Egg Safety and Quality Research Unit US National Poultry Centre Agricultural Research Service U.S. Department of Agriculture Athens Georgia

**Keywords:** angel food cake, cake meringue, foaming properties, mixing, pasteurized eggs

## Abstract

Due to recent *Salmonella* outbreaks, the pasteurized shell egg market is rapidly growing. One objection to using pasteurized eggs is the belief that they will produce unacceptable angel food cakes. Eggs were pasteurized using a hot water immersion process (56.7°C for 60 min) similar to that used by industry. Angel food cakes were made from the pasteurized egg white (PEW) as well as from raw egg white (REW) for comparison. Meringues were made using three mixer speed settings (low, medium, and high) and three durations for each speed. Functional qualities such as egg foaming were evaluated. Angel food cakes were compared in terms of cake volume, texture profile, and color values. When the optimal processing factors used for REW were applied to PEW, an inferior meringue was formed. However, by increasing the mixing time for PEW by 200% at the highest speed, an acceptable meringue was formed. The best angel food cake prepared from PEW had a volume only 6.8% less than that of the best cake prepared from REW. Texture profile analyses showed that the best angel food cake made from PEW was 13% firmer, 7.4% less springy, and 62% chewier than that from REW. Color analyses showed that PEW made a slightly darker colored cake crust than REW, although there were no significant differences in the crumb color. Modifying the mixing conditions for PEW resulted in angel food cakes with quality similar to that of cakes made with REW, thus overcoming an objection to using safer pasteurized shell eggs.

## 1 INTRODUCTION

Eggs contain a high amount of proteins, which undergo denaturation on the application of heat treatment. Pasteurized shell eggs are heated at time–temperature combinations which affect the functionality of eggs. Due to recent *Salmonella* outbreaks, the number of eggs that are commercially pasteurized is increasing (Geveke, Gurtler, Jones, & Bigley, 2016).

Egg white is composed of a variety of proteins that range widely in chemical properties (molecular weight, pI, glycosylation, phosphorylation, and sulfhydryl/disulfide content) (Li‐Chan & Nakai, 1989). Egg whites are used in many food formulations due to their foaming, emulsification, and leavening ability. This foaming ability contributes toward improving the structure/texture of batters and baked products (Singh & Ramaswamy, 2013; Van der Plancken, Van Loey, & Hendrickx, 2005). The quality of these products is mainly determined by the foaming properties of egg white. When egg white is whipped, air bubbles are trapped in liquid albumen which causes an increase in the foaming volume of the egg white. Nakamura (1963) observed that ovomucin, globulins, and conalbumin have high foaming power, whereas lysozyme, ovomucoid, and ovalbumin exhibit low foaming characteristics.

Egg white is one of the main ingredients used in the production of angel food cakes, meringues, and soufflés. Not all individual egg white proteins have the same functional ability in the production of angel food cake. Ovalbumin (54% of total protein) and globulins (8% of total protein), when tested individually, are the two major proteins that help in producing angel food cakes with volume equal or greater than egg white (Johnson & Zabik, 1981a,b; Li‐Chan & Nakai, 1989). Functional properties of albumen have been discussed in detail by many authors (Abeyrathne, Lee, & Ahn, 2013; Jones, 2007; Raikos, Campbell, & Euston, 2007; Singh & Ramaswamy, 2014; Singh, Sharma, & Ramaswamy, 2015) including a comprehensive review by Mine and Zhang (2002).

Angel food cake preparation is utilized to determine the foaming and leavening capability of albumen (Pernell, Foegeding, Luck, & Davis, 2002). Angel food cake is of particular interest among food foam products because the protein foam is generated independently and then combined with other ingredients, allowing for separate investigation of the properties of the foam and food product. In essence, it serves as a model system for investigating foam formation and the thermal transformation from a fluid to a solid foam (Berry, Yang, & Foegeding, 2009).

Angel food cakes made with whey protein foams have lower volume than similar cakes made with egg white foams (Arunepanlop, Morr, Karleskind, & Laye, 1996; Berry et al., 2009; Mohamed, Lajis, & Hamid, 1995; Pernell, Luck, AllenFoegeding, & Daubert, 2002). This shows that egg white proteins play an important role in making good quality angel food cake. Heating/pasteurization of egg due to *Salmonella* and other food safety issues may damage these proteins, and these changes may lead to a poor quality angel food cake. Regarding pasteurized liquid egg whites, an addition of whipping aids or additional beating time is required to restore the foaming ability (Johnson & Zabik, 1981b; Kline, Sugihara, & Ijichi, 1966). Thermal heating of the eggs decreases the foaming ability of the eggs and increasing mixing speed can improve foaming (Kline et al., 1966), but very limited research is available about the effects of mixing speed and time on the foaming, and functionality of meringues and angel food cakes. No studies exist on improving the quality of angel food cakes made from egg whites from pasteurized shell eggs.

The current study was conducted to examine how the functionality of whites from pasteurized shell eggs can be enhanced by using additional mixing. Longer mixing times were correlated to the final product, that is, angel food cake. This correlation can be used to study the impact of additional beating on egg foaming and related products such as egg white meringues. The question of whether an acceptable angel food cake could be made from pasteurized shell eggs was investigated.

## 2 MATERIALS AND METHODS

### 2.1 Preparation of egg whites from pasteurized shell eggs

Eggs were acquired from an industrial partner (Esbenshade Farms, Mount Joy, PA, USA). Pasteurized egg white (PEW) and raw egg white (REW) were used to prepare foams. Egg white was separated from egg yolk by using a Tovolo brand silicon yolk separator. The separator drew the yolk out of the whole egg which had been spread out on a flat surface. In the present study, shell eggs were pasteurized at 56.7°C for 60 min using a Thermo hot water circulating bath (Geveke, Bigley, Brunkhorst, Jones, & Tilman, 2018). This process is representative of the commercial pasteurization process used by industry. Geveke et al. (2018) determined that the population of *Salmonella* in shell eggs was reduced from 6.1 ± 0.1 log CFU/ml to 1.1 ± 0.5 log CFU/ml following a 56.7°C water bath treatment for 60 min.

### 2.2 Determination of foaming

Foaming was accomplished by adding 50 ml (20°C) egg white into a 400‐ml beaker followed by mixing (Waring WSM7Q 7 qt. Commercial NSF Stand Mixer – 120V, 1 HP) for different time and speed combinations as described in Table 1. Foam volume was measured using the procedure of Raikos et al. (2007).

**Table 1:** Combination of mixing speeds and mixing times employed in preparing foams and angel food cake meringues (n = 3)

Egg Type	Speed	Time (min)
Raw	6, 9, 12	1, 2, 3, 4
Pasteurized	6, 9, 12	6, 8, 10, 12

### 2.3 Meringue preparation

For the angel food cake meringue preparation, a modified version of the Jones (2007) procedure was used. The procedure was adjusted accordingly to accommodate the mixer and oven utilized. Egg whites from shell eggs were brought to room temperature (approximately 20°C) and were mixed using an automatic mixer (Waring WSM7Q) capable of speeds up to 12. The following recipe was used to make the meringue: liquid egg whites (100 ml), cake flour (33 g), salt (1/8 teaspoon), cream of tartar (1/4 teaspoon), and sugar (65 g). To make the meringue, liquid egg whites, salt, and cream of tartar were mixed for 30 s which was sufficient to initiate the foaming process. Once the foam started to form, the sugar and 1/3 of the cake flour were added while mixing at the different speed and time combinations as presented in Table 1. The sugar and cake flour were added slowly to allow proper mixing and to prevent any grittiness and lump formation in the meringue. Upon completion of the mixing step, the rest of the cake flour was folded into the mixture using a spatula type spoon.

### 2.4 Angel food cake preparation

Angel food cake was prepared by pouring 75 ± 2 g of meringue into each 1 pound size loaf pan (8.57 × 12.5 × 6.35 cm) (Jones, 2007). Angel food cakes were baked at 350°F (176°C) for 32 min in a convection oven (Avantco CO‐16 Half Size Countertop) bought from Webstaurant store (2205 Old Philadelphia Pike, Lancaster, PA 17602). All cakes were inverted and allowed to cool for 1 hr. Angel food cakes were measured for height, volume, color, texture, and weight loss. Angel food cakes were stored in airtight Ziploc^™^ bags at room temperature for 24 hr to measure weight loss of the cake (Jones, 2007).

### 2.5 Angel food cake volume and height determination

Cake volume was measured by using a seed displacement method (Segnini, Pedreschi, & Dejmek, 2004). Rapeseeds were put in the empty loaf pan to measure the total volume. After baking the angel food cake in the loaf pan, rapeseeds were put in to fill the empty volume of the loaf pan. By measuring the total volume and unoccupied volume, the volume occupied by the angel food cake was obtained. The final height for each formulation was also determined. Angel food cakes were measured from the base to its highest point as per the procedure of Martínez‐Cervera, Sanz, Salvador, and Fiszman (2012).

### 2.6 Determination of weight loss

After baking, the initial weight (*W*_*i*_) in g for each cake was recorded. After baking and the 24 hr storage period, the final weight (*W*_*f*_) was measured. Total weight loss (%) was calculated by using the following formula:

$$\%\;\text{Weight loss}=\left(W_{i}-W_{f}\right)/W_{i}\times 100$$

### 2.7 Texture profile analysis determination

Angel food cakes were tested in detail for changes in their texture profile analysis (TPA). The texture of the angel food cakes was expressed in terms of hardness, springiness, and gumminess. A texture analyzer with a 5 kg load cell (TA.XT plus, Texture Technologies Corp., Scarsdale, NY, USA) was used to evaluate the TPA. The TA.XT plus software was used for measuring the following parameters:

1. Hardness: the maximum force required to compress the sample,
2. Springiness: the height that the food recovers during the time that elapses between the end of the first bite and the start of the second bite,
3. Chewiness: the energy required to chew a food to the point required to swallow it (applies only to solid products), and
4. Resilience: the power of the product to fight back to its original height. Resilience is a measurement of how a sample recovers from deformation in relation to speed and forces derived. It is calculated by dividing the upstroke energy of the first compression by the down stroke energy of the first compression.

### 2.8 Color measurement

The surface color characteristics of angel food cake crust (outer layer) and crumb (inner layer next to the crust) samples were evaluated in L, a, and b units using a tristimulus Minolta CM‐508d spectrophotometer (Minolta Co., Japan). The instrument was warmed up for 10 min before the actual measurements, and calibration was performed using a white standard plate. Multiple measurements (6) were made individually for each sample, and the average value was reported. The color values of the crust and crumb samples were determined in a three‐dimensional color space, L (luminosity), a (green − to red +), and b (blue − to yellow +).

### 2.9 Statistical analysis

Mathematical and statistical analysis was performed on the data using analysis of variance (ANOVA) with the help of Minitab 16 Statistical Software (Minitab Inc., State College, PA, USA). ANOVA analysis was done, and the significance level was reported with 95% level of confidence. The analysis was performed to evaluate the effect of treatment factors (mixing speed and time) on the functionality.

## 3 RESULTS AND DISCUSSION

### 3.1 Foaming capacity of eggs

**Figure 1:**
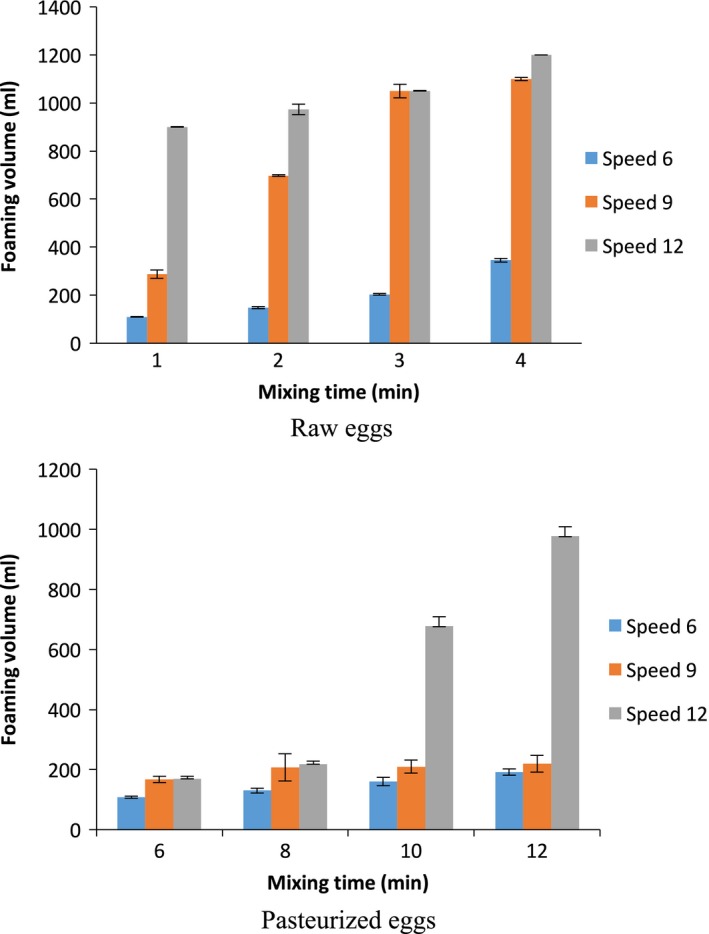
Foaming volume of egg whites from raw eggs and pasteurized eggs at various mixing speeds/times (*n* = 3)

Unpasteurized REWs are known to have excellent foaming properties. Egg whites were whipped using the experimental setup as described in the materials and methods section. Increasing both mixing time and speed resulted in the formation of higher volume foams (Figure 1a) as expected with raw eggs. Saturation occurred at mixing speed 12 with times of 3 and 4 min. Foaming was found to be significantly (*p *< 0.05) affected by both mixing time and mixing speed. In raw eggs, by doubling the mixing speed from 6 to 12 at 1 min, the foaming increased from 109 to 900 ml (almost nine times). This trend was also observed by increasing mixing times; for instance, increasing the mixing time from 1 to 4 min, at speed 6, increased foaming from 109 to 345 ml. A significant difference (*p *< 0.05) was observed in the foam volume of REW prepared by using mixing times of 1, 2, 3, and 4 min at each speed of 6, 9, and 12.

Overall, egg whites from raw unpasteurized shell eggs had higher foam volumes than from the pasteurized shell eggs (Figure 1b). The mixing times used for the raw eggs (1, 2, 3, and 4 min) led to very little foaming of the pasteurized eggs, so longer mixing times (6, 8, 10, and 12 min) were employed for the pasteurized eggs. Due to thermal protein denaturation in the pasteurized eggs, the foaming values were relatively lower than the raw eggs. For instance, by increasing the mixing speed from 6 to 12 at 6 min, the foaming increased from 107 to 170 ml which is comparatively smaller than that of the raw eggs. But, when the mixing time was increased from 6 to 12 min, at a speed of 12, foaming values increased from 170 to 977 ml. This indicates that extended mixing times lead to higher foaming values using pasteurized eggs. It must be noted that substantial amounts of foam from raw eggs were obtainable at both speeds 9 and 12, whereas adequate foams from pasteurized eggs could only be achieved at speed 12. It has been known that by increasing the mixing/beating time by a factor of 2 or more can overcome the effect of pasteurizing liquid egg white product (Kline, Sugihara, Bean, & Ijichi, 1965). The results of the present study on whites from pasteurized shell eggs agree with those on pasteurized liquid egg white product.

A major component of angel food cake meringue is egg white, so it suggests that longer mixing times of egg white can aid in meringue formation from pasteurized shell eggs. While evaluating these foaming properties of egg whites, it is important to keep in mind that during foam formation, the proteins undergo additional denaturation at the interface (Singh & Ramaswamy, 2013, 2015; Singh et al., 2015). It has been known since the 1960s that pasteurization of liquid egg white product decreases the foaming ability, and increasing the mixing time can improve foaming (Kline et al., 1966; Singh, 2012), but very limited research is available about the effects of mixing speed and time on the foaming, and functionality of meringues and angel food cakes. Overall, the results of the present study determined that using a combination of longer times at the highest mixing speed improved the foam volume from the pasteurized shell eggs, but it was still 19% less than that from raw eggs.

**Table 2:** Total volume of angel food cakes made from raw and pasteurized egg whites (n = 3)

Time (min)	Speed 6	Speed 9	Speed 12
	Volume (ml)	Volume (ml)	Volume (ml)
Raw eggs			
1	295.0 ± 26.4^c^	232.5 ± 3.5^a^	312.5 ± 14.1^c^
2	255.0 ± 16.0^b^	265.0 ± 7.0^b^	335.0 ± 17.7^c^
3	265.0 ± 15.0^b^	355.0 ± 21.2^d^	370.0 ± 12.9^d^
4	n/a	322.5 ± 3.5^c^	355.0 ± 4.9^d^
Pasteurized eggs			
6	210.0 ± 7.0^a^	255.5 ± 7.7^b^	327.6 ± 3.0^d^
8	205.0 ± 26.5^a^	275.0 ± 39.0^c^	344.7 ± 18.0^d^
10	203.3 ± 16.0^a^	332.5 ± 10.6^d^	331.5 ± 9.4^d^
12	255.0 ± 40.0^b^	312.5 ± 10.6^d^	341.0 ± 13.5^d^

The superscript letters (a, b, c, d) represents that means within columns with similar letters are not significantly different (*p *>* *0.01).

### 3.2 Angel food cake volume and height

Cake volume and height are some of the most important characteristics which tell the true tale of cake quality. Angel food cakes volume and height generally increased with increasing mixing time at each mixing speed. But, the volume increase in the cakes made from the raw eggs was greater than that made from the pasteurized shell eggs. The maximum cake volumes with REW were obtained at speed 12 (3 and 4 min mixing time) as seen in Table 2. Mixing times of 3 and 4 min did not yield satisfactory angel food cakes with PEW, so optimized (longer) mixing times of 6, 8, 10, and 12 min were employed. Longer mixing times help form better structural networks and thus compensate for the denatured egg protein during the pasteurization. Even with the extended mixing times, pasteurized egg angel food cakes had slightly lesser volumes than the raw egg angel food cakes. Using pasteurized eggs, the largest cake volumes (331–345 ml) were found at a mixing speed of 12 with times of 8–12 min. These volumes were similar to the cakes formed from REW at the mixing speeds of 9 and 12. The highest cake volumes using raw eggs at speeds 9 and 12 were 355 and 370 ml, respectively (Table 2). This shows that even with the pasteurized shell eggs, angel food cake volumes similar to those made with REW can be obtained using modified mixing conditions.

**Figure 2:**
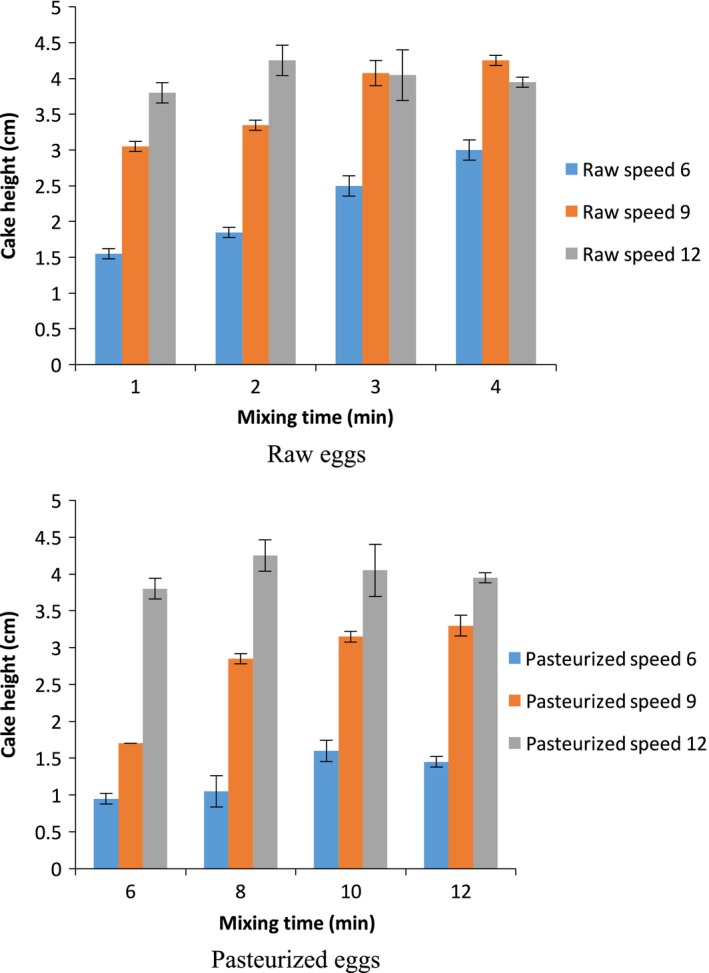
Height (cm) of angel food cake made from raw eggs and pasteurized eggs at various mixing speeds/times (*n* = 3)

The mixing conditions (mixing speed and time) also had a significant effect on the angel food cake height. Figure 2 shows an increase in cake height as mixing time increased using raw eggs. The crust structure of baked angel food cakes provides an explanation. Increased mixing time for angel food cake made from both raw and PEWs resulted in increased prevalence of large air cells. Longer mixing decreased the extent and rate of gluten polymerization during heating (Van Steertegem, Pareyt, Brijs, & Delcour, 2013).

Angel food cakes height significantly (*p *< 0.05) changed with increasing mixing speed for pasteurized eggs (Figure 2). At speed 12, cake height was very similar for both raw and pasteurized eggs. At mixing speeds 6 and 9, maximum heights of angel food cake made from REW were 1.85 and 4.07 cm, respectively, which were higher than the maximum heights of cakes made using pasteurized eggs (i.e., 1.6 and 3.15 cm, respectively). But, at speed 12, the maximum height obtained using raw eggs was 4.25 cm which was not significantly different (*p *< 0.05) from that of pasteurized eggs which was 4.22 cm. This shows that at speed 12, pasteurized eggs can form higher quality meringue which can in turn achieve the same angel cake height as that of the raw eggs. So, using the highest mixing speed and longer mixing times improves the functionality of pasteurized shell eggs for making angel food cake.

During extensive mixing, the properties of the gluten polymer network presumably change in such a way that it exerts a further effect on subsequent polymerization during baking. More so, the resulting glutenin structure incorporates less gliadin in the polymer network during baking than when mixed for shorter durations. This can probably be related to an altered availability (due to conformational changes) of the sulfhydryl (SH) groups. (Van Steertegem et al., 2013). This may be due to gluten depolymerization (Tanaka & Bushuk, 1973; Weegels, Hamer, & Schofield, 1997) or to (partial) unfolding of gluten proteins initially present in the flour particles.

### 3.3 Cake weight loss

Weight loss is one of the important functionality characteristics. It is an indicator which reveals the postbaking changes in moisture and related changes in the volume and texture. A rapid moisture loss could lead to a cake with reduced volume and a crumbly texture. So, postbaking conditions are important for a high‐quality final product.

**Figure 3:**
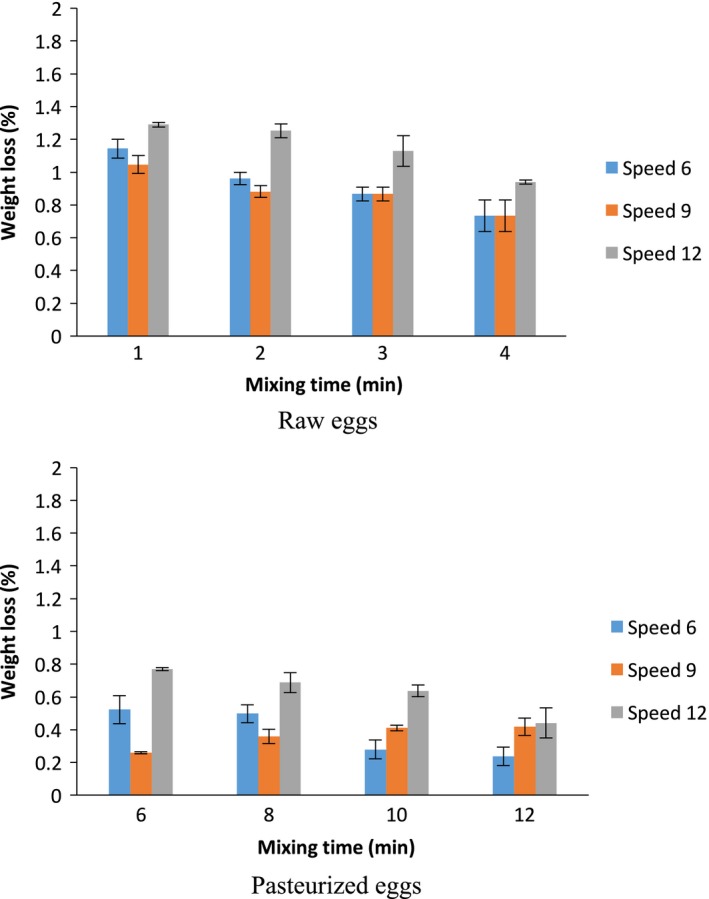
Weight loss of stored angel food cakes made from raw egg whites and pasteurized egg whites (*n* = 3)

The angel food cake samples were stored in polyethylene bags for 24 hr to observe changes in the weight. Weight losses of angel food cakes made from REW were different (*p *> 0.05) from those from PEW. In both cases, more weight loss happened at the highest mixing speed and lowest time, that is, 12 speed and 6 min for pasteurized eggs and 1 min for raw eggs. Values for cake weight loss (%) are found in Figure 3. The highest weight losses from the cakes with raw egg at each mixing speed (6, 9 and 12) were found to be 1.14, 1.04, and 1.29%. However, from the cakes made with the pasteurized eggs, weight loss was found to be 0.7, 0.41 and 0.76%, which is almost half of that of the raw eggs. This could be due to the fact that PEW cakes had a greater hardness and a less porous structure than REW cakes (as discussed in the TPA section).

### 3.4 Texture profile analysis

#### 3.4.1 Hardness

Hardness is defined as the peak force during the first compression cycle by a texture analyzer. It is a mechanical measure of the energy exerted upon biting a cake and is indicative of the firmness of the cake. The hardness of angel food cakes made from pasteurized and REWs was compared. Angel food cakes are generally soft in texture, and lower hardness values are desirable. When angel food cakes were made with raw eggs, hardness was found to decrease with increasing mixing speed and mixing time. Using raw eggs, at speed 6, the meringue was not fully formed, which led to hard cakes but speeds 9 and 12 were sufficient to fully form a meringue which resulted in soft and spongy cakes (decreased hardness values in Table 3a). Lower mixing times led to hard cakes as meringue was not formed completely, and a hard cake structure was made during baking. For example, the hardness of the angel food cakes from the raw eggs decreased from 509 (3 min) to 449 (4 min) at speed 6. Cakes were texturally hard with a hardness value of around 500 at a mixing speed of 6, but hardness decreased to 405 (3 min) and 327 (4 min) at speed 9 (Table 3). Angel food cakes with hardness values of less than 400 were found to have a soft desirable texture.

**Table 3:** Texture profile analyses of angel food cakes obtained from raw and pasteurized egg whites (n = 3)

Speed/ time	Hardness	Resilience	Springiness	Chewiness
Raw eggs				
Speed 6/3 min	508.9 ± 19.2^d^	34.2 ± 3.3^d^	83.3 ± 1.6^d^	314.7 ± 20.2^d^
Speed 6/4 min	448.9 ± 15.1^d^	37.3 ± 2.9^d^	84.9 ± 8.1^d^	333.1 ± 15.3^d^
Speed 9/1 min	352.2 ± 11.9^a^	30.6 ± 1.2^c^	77.6 ± 1.8^d^	181.3 ± 6.2^b^
Speed 9/2 min	425.1 ± 18.6^cb^	25.9 ± 1.1^c^	72.4 ± 3.4^c^	181.7 ± 13.4^b^
Speed 9/3 min	405.3 ± 25.4^b^	22.9 ± 2.1^a^	70.6 ± 3.8^c^	161.2 ± 13.2^b^
Speed 9/ 4 min	327.3 ± 23.4^a^	24.8 ± 2.4^b^	68.0 ± 3.9^b^	127.9 ± 11.1^a^
Speed 12/ 1 min	381.9 ± 29.5^b^	26.8 ± 3.7^c^	87.0 ± 8.6^d^	265.8 ± 18.8^c^
Speed 12/ 2 min	330.6 ± 11.8^a^	23.1 ± 1.8^b^	68.6 ± 3.8^b^	125.0 ± 13.5^a^
Speed 12 / 3 min	388.4 ± 05.6^b^	21.1 ± 1.6^a^	70.4 ± 3.6^c^	147.4 ± 18.7^ab^
Speed 12/4 min	462.4 ± 20.5^d^	23.4 ± 3.1^b^	74.3 ± 2.1^c^	218.0 ± 24.2^c^
Pasteurized eggs				
Speed 6/ 6 min	343.0 ± 33.8^a^	16.2 ± 1.9^a^	58.2 ± 3.9^ab^	155.8 ± 13.3^a^
Speed 6/8 min	325.8 ± 19.5^a^	20.5 ± 2.6 ^b^	65.3 ± 3.9^b^	167.8 ± 20.2^a^
Speed 6/ 10 min	951.2 ± 26.6^d^	30.0 ± 1.1^d^	45.2 ± 8.0^a^	647.8 ± 28.3^d^
Speed 6/ 12 min	516.6 ± 21.6^c^	28.8 ± 2.0 ^d^	71.9 ± 2.0^c^	319.3 ± 24.1b
Speed 9 / 6 min	548.3 ± 76.0^c^	26.4 ± 1.9^c^	71.8 ± 2.0^c^	347.3 ± 14.1^b^
Speed 9 / 8 min	690.4 ± 74.7^c^	25.5 ± 1.3^c^	73.6 ± 3.0^d^	443.2 ± 22.0^c^
Speed 9/10 min	508.2 ± 20.0^b^	26.4 ± 0.8^c^	73.2 ± 2.8^d^	308.3 ± 11.6^b^
Speed 9/12 min	518.6 ± 40.7^b^	28.3 ± 1.8^d^	74.9 ± 3.7^d^	323.7 ± 20.5^b^
Speed 12/6 min	516.6 ± 21.6^b^	26.8 ± 2.0^c^	71.8 ± 2.0^c^	319.3 ± 24.1^b^
Speed 12/8 min	371.0 ± 24.4^a^	26.6 ± 1.0^c^	68.8 ± 1.8^b^	335.4 ± 11.9^b^
Speed 12/10 min	382.4 ± 7.7^ab^	20.8 ± 4.0^b^	62.5 ± 3.7^b^	202.9 ± 5.3^a b^
Speed 12/12 min	462.6 ± 22. 3 b	21.4 ± 2.2^b^	66.7 ± 3.3^b^	247.1 ± 20.5^ab^

The superscript letters (a, b, c, d) represents that means within columns with similar letters are not significantly different (*p *>* *0.01).

Similar trends were found with the cakes made from the PEW. When the same speed and time combinations were used for pasteurized eggs as that of the raw eggs, the meringues were not fully formed, so mixing times were increased to 6, 8, 10, and 12 min at the same speeds (6, 9, and 12). When these mixing times were used at mixing speeds of 9 and 12, acceptable quality meringues were formed. However, only at speed 12 were cakes of desirable hardness obtained using PEW. These were relatively soft and nearly comparable to the cakes made from the REW. At speeds 6 and 9, hardness was unacceptable (Table 3). Using PEWs, cakes with a hardness of 371 were formed using a mixing speed 12 for 8 min. The softest cakes were produced at speed 12 for both pasteurized and raw eggs.

#### 3.4.2 Springiness

Springiness is the ability of a product to spring back (recover) after deformation during first compression. Spring back is measured with a texture analyzer at the down stroke of the second compression, so duration between the two compressions is very important. Low springiness would lead to a soggy structure of the angel food cake, so high springiness is desirable. The springiness is very important in angel food cakes as it influences the texture and acceptability of food. The mixing times and speeds influenced the batter structure in such a way that led to changes in the cakes’ springiness.

When baking is done to set a good quality product, the outward pressure from bubble expansion cannot exceed the rate of the protein coagulation and starch gelatinization (Pateras, Howells, & Rosenthal, 1994). If the bubble pressure is too great, then bubbles coalesce and escape the product before a strong matrix forms. The resultant baked product either deflates or has reduced volume. The cake quality is dependent on its hardness and springiness. More or less mixing can form an optimal gluten network which will help in forming a good structure during baking. Optimal mixing can produce a good crumb and crust structure. It also allows CO_2_ retention, which increases the potential for an ideal gluten network.

Overall, the angel food cakes made from pasteurized eggs were less springy than the angel food cakes made from the raw eggs at mixing speed 12 (Table 3). The average springiness of the angel food cakes made from raw eggs was 75 versus 67 for cakes from pasteurized eggs. The texture analysis results indicate that the angel food cakes from raw eggs were springier than from pasteurized eggs.

#### 3.4.3 Chewiness

This property applies only to solid products and is calculated by gumminess multiplied by springiness (which is Distance2/Distance1 obtained from the texture analyzer). Chewiness is an important measurement as a too chewy angel food cake would not be acceptable. A cake with high chewiness value requires more chewing ability. The TPA results showed a decreasing trend in chewiness with increasing mixing times for both raw and pasteurized eggs (Table 3). Generally, raw egg angel food cakes were less chewy than pasteurized cakes. Angel food cakes made from raw eggs at a mixing speed of 12 gave an average chewiness value of 189 versus 276 for pasteurized egg angel food cakes.

#### 3.4.4 Resilience

Resilience is calculated by dividing the upstroke energy of the first compression by the down stroke energy of the first compression as calculated by the TPA software. Resilience is how well a product “fights to regain its original height.” Angel food cake made from the raw eggs had higher resilience than the cakes from the pasteurized eggs. The highest resilience values from the cakes with raw eggs at mixing speeds of 6, 9, and 12 were 37.3, 30.6, and 26.8, respectively, which were relatively higher than the pasteurized cakes. The pasteurized egg cakes had resilience values of 30, 28.8, and 26.8 at the same mixing speeds. The resilience was found to decrease with increasing mixing speed for both raw and pasteurized eggs. Overall, pasteurized egg angel food cakes were less resilient than the raw egg angel food cakes.

Overall, comparing the textural properties of angel food cakes made from raw eggs and pasteurized eggs, it was found that there were differences. Angel food cakes from the raw eggs were slightly softer, less chewy, and more resilient and springy than those from the pasteurized eggs.

### 3.5 Variation in color

**Figure 4:**
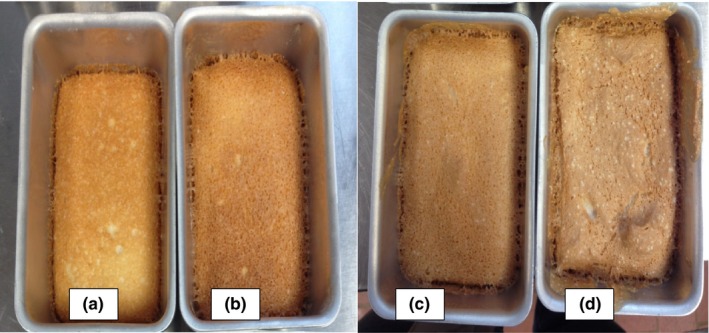
Angel food cakes from raw egg whites with mixing speed 9 and mixing times of (a) 1 min, (b) 2 min, (c) 3 min, and (d) 4 min. (*n* = 3)

**Figure 5:**
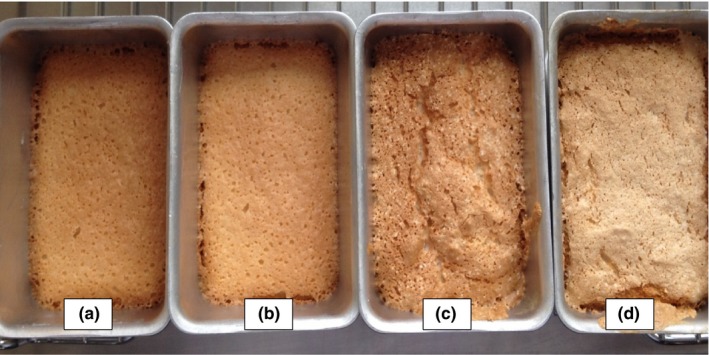
Angel food cakes from pasteurized egg whites with mixing speed 12 and mixing times of (a) 6 min, (b) 8 min, (c)10 min, and (d) 12 min. (*n* = 3)

The interior of an angel food cake, the crumb, is known for its pristine whiteness. Changing the mixing speeds and times led to variations in the appearance of the crust, but not of the crumb. Longer mixing times led to darker angel food cake crusts and a visible increase in the volumes of the cakes (Figures 4 and 5). Cakes made from pasteurized eggs gave a darker appearance than that of the cakes from raw eggs.

Mixing time and mixing speed had significant effects on the color characteristics of the crust (Table 4). The L values (lightness/brightness) generally decreased with increasing mixing times at speed 12 in agreement with the observations made based on Figures 4 and 5. L values for the angel food cake crust from REW averaged 61 at mixing speed 12. When angel food cakes were made with PEW at the same speed, the L values averaged 59.

The redness/green “a” value acts as an indicator of the reddish to green color in the sample. The “a” values decreased with increasing mixing times at speed 12. Angel food cake crusts from REW resulted in “a” values averaging 7.9 at mixing speed 12. For angel food cakes made from PEW at the same speed, the “a” values averaged 9.0.

The “b” (yellowness or blueness) values showed a somewhat similar trend to those of the L and “a” values; the “b” values generally decreased with increasing mixing times at speed 12. Angel food cake crusts from both REW and PEW resulted in “b” values averaging 21 at mixing speed 12.

**Table 4:** Crust color characteristics of angel food cakes obtained from raw eggs and pasteurized eggs(n = 3)

Speed	Time	L	a	B
Raw eggs				
6	1	n/a	n/a	n/a
	2	n/a	n/a	n/a
	3	58.9 ± 2.1 ^c^	5.6 ± 0.5^a^	20.7 ± 1.7^d^
	4	53.8 ± 2.8^b^	12.1 ± 0.8^d^	21.8 ± 0.78^d^
9	1	n/a	n/a	n/a
	2	42.9 ± 6.0^a^	9.5 ± 0.7^c^	17.5 ± 1.4^a^
	3	62.4 ± 3.5^d^	8.5 ± 0.9^c^	22.9 ± 0.5^d^
	4	57.3 ± 6.0^b^	6.8 ± 0.8^b^	18.8 ± 1.8^b^
12	1	54.5 ± 3.3^b^	9.1 ± 0.7^c^	22.6 ± 1.6^d^
	2	60.5 ± 5.3^c^	9.1 ± 0.7^c^	22.6 ± 1.6^d^
	3	64.5 ± 6.9^d^	6.83 ± 0.1^b^	18.9 ± 1.2^b^
	4	57.8 ± 5.9^b^	6.41 ± 0.4^b^	18.2 ± 1.1^b^
Pasteurized eggs				
6	6	55.6 ± 1.82^a^	8.22 ± 0.97^b^	22.45 ± 1.30^b^
	8	59.29 ± 3.67^c^	8.80 ± 1.12^b^	22.81 ± 1.10^b^
	10	59.29 ± 3.67^c^	8.80 ± 1.12^b^	22.81 ± 1.10^b^
	12	59.29 ± 3.67^c^	8.80 ± 1.12^b^	22.81 ± 1.10^b^
9	6	54.72 ± 2.30^a^	11.29 ± 1.10^d^	24.56 ± 1.70^d^
	8	58.85 ± 3.00 ^c^	9.23 ± 0.51^c^	22.31 ± 0.45^c^
	10	59.6 ± 2.45^c^	8.94 ± 0.34^b^	21.17 ± 1.57^b^
	12	53.74 ± 2.44^a^	12.61 ± 0.56^d^	25.64 ± 1.49^d^
12	6	63.83 ± 1.71^d^	10.99 ± 0.97^c^	23.18 ± 1.54^c^
	8	59.26 ± 1.23^c^	10.91 ± 1.03^c^	22.70 ± 0.16^b^
	10	57.31 ± 4.60^b^	6.99 ± 0.76^a^	18.87 ± 1.13^a^
	12	56.89 ± 3.81^b^	6.94 ± 0.54^a^	19.21 ± 1.09^a^

The superscript letters (a, b, c, d) represents that means within columns with similar letters are not significantly different (*p *>* *0.01).

Overall, from the color value analysis of L, a, and b values, it was observed that there were slight differences in lightness and redness, but not yellowness, of the angel food cake crusts made from the pasteurized and raw eggs. Angel food cakes from pasteurized eggs had lower L values and higher “a” values than those from raw eggs.

## 4 CONCLUSIONS

It can be concluded that by modifying the processing factors of mixing meringue, acceptable quality angel food cakes can be made with pasteurized shell eggs. Additional mixing time was required to make a satisfactory angel food cake using pasteurized eggs. In general, raw eggs made slightly better quality angel food cakes than the pasteurized eggs. PEW produced less egg foam volume, as well as harder and chewier angel food cakes than did REW. However, angel food cakes from pasteurized eggs had similar crumb and crust color, cake volume and height, as well as cake springiness and resilience to those of cakes made from raw eggs. Overall, acceptable quality angel food cakes can be made from safer pasteurized shell eggs.

## CONFLICT OF INTEREST

The authors declare that they do not have any conflict of interest.

## ETHICAL STATEMENTS

*Ethical review:* This study does not involve any human or animal testing.

*Informed consent:* This study was not performed on any participants.
